# A17 SYSTEMS-LEVEL PATHWAY MAPPING REVEALS GUT–BRAIN AND IMMUNE SIGNATURES IN FLARES OF IBS

**DOI:** 10.1093/jcag/gwaf042.017

**Published:** 2026-02-13

**Authors:** V Mohan, M Pinto-Sanchez, A Nardelli, M Magee, R Borojevic, Z Kroezen, M Shanmuganathan, G De Palma, P Moayyedi, P Britz Mckibbin, S Collins, P Bercik

**Affiliations:** Department of Medicine, McMaster University, Hamilton, ON, Canada; Department of Medicine, McMaster University, Hamilton, ON, Canada; Department of Medicine, McMaster University, Hamilton, ON, Canada; McMaster University, Hamilton, ON, Canada; Department of Medicine, McMaster University, Hamilton, ON, Canada; McMaster University, Hamilton, ON, Canada; McMaster University, Hamilton, ON, Canada; Department of Medicine, McMaster University, Hamilton, ON, Canada; Department of Medicine, McMaster University, Hamilton, ON, Canada; McMaster University, Hamilton, ON, Canada; Department of Medicine, McMaster University, Hamilton, ON, Canada; Department of Medicine, McMaster University, Hamilton, ON, Canada

## Abstract

**Background:**

Irritable bowel syndrome (IBS) is a disorder of gut-brain interaction affecting an estimated 5.8% of Canadians (Rome IV criteria). Although accumulating evidence implicates the gut microbiota in IBS pathophysiology, identifying the key drivers of symptom expression remains challenging due to pronounced interindividual variability. We hypothesized that individualized microbiota–metabolite interactions may drive distinct host pathway responses underlying symptom variability during flares.

**Aims:**

To identify signaling pathways underlying flare-associated microbiota–metabolite signatures in IBS patients.

**Methods:**

Subjects with microbiota (16S rRNA sequencing) and metabolite (untargeted metabolomics) alterations linked to symptom flares were identified from a 25-week longitudinal cohort of 28 IBS patients (∼950 samples). For each subject, altered metabolites were integrated with microbial associations and gene targets to generate individualized datasets. Gene targets were annotated with Entrez IDs, and KEGG pathway enrichment (p < 0.05) was performed using *clusterProfiler*, retaining the top 10 pathways. Multi-partite networks were visualized in *igraph*.

**Results:**

Microbiota–Metabolite–Gene–Signaling Pathway (MMGS) networks were generated for ten IBS subjects showing significant correlations between microbiota shifts and symptom fluctuations over time. Metabolites occupied the central node position, representing their dual microbial and host origins. The Neuroactive ligand–receptor interaction pathway was the most frequently altered (9/10 subjects), consistent with gut–brain axis involvement in IBS. The Glutamatergic synapse pathway was identified in 7/10 subjects, while Phospholipase D signaling and Neutrophil extracellular trap (NET) formation pathways appeared in 5/10 subjects each. Subtype-related trends such as: Arginine biosynthesis, Circadian entrainment, and Dopaminergic synapse were more prevalent among IBS-C subjects, whereas Glutamatergic synapse and cAMP signaling dominated in IBS-D. Notably, in two IBS-D subjects, infection- and inflammation-related pathways and NET formation, respectively, were the *only* pathways identified, suggesting distinct immune-driven mechanisms underlying their symptom flares.

**Conclusions:**

Pathway incidence profiling showed that subjects clustered independently of clinical subtype, suggesting that pathway-based signatures may better capture the mechanistic heterogeneity in IBS than symptom-based classifications. The MMGS network highlights both shared and individual pathway perturbations, supporting individualized profiling to define mechanistic subgroups, advancing precision-based approaches for IBS management.

**Funding Agencies:**

CIHR

